# Objective Sleep, 24‐hour Rest‐Activity Rhythms, and Cognition

**DOI:** 10.1002/alz70860_100553

**Published:** 2025-12-23

**Authors:** Clémence Cavaillès, Katie L Stone, Yue Leng, Carrie B. Peltz, Kristine Yaffe

**Affiliations:** ^1^ University of California, San Francisco, San Francisco, CA, USA; ^2^ California Pacific Medical Center Research Institute, San Francisco, CA, USA; ^3^ San Francisco Veterans Affairs Health Care System, San Francisco, CA, USA

## Abstract

**Background:**

Poor sleep and circadian disruptions have been associated with cognitive decline and increased dementia risk. However, limited research has explored the relationship between objective sleep measures, circadian rest‐activity rhythms (RAR), and specific cognitive domains.

**Method:**

We analyzed data from 856 participants aged ≥50 years in the Dormir Study. Objective nighttime sleep metrics (sleep duration, sleep efficiency, wake after sleep onset (WASO), and sleep fragmentation index (SFI)), along with four RAR parameters from the extended cosine model (amplitude, mesor, acrophase, and pseudo‐F) were derived from 7‐day wrist actigraphy. We examined the associations between actigraphy‐derived variables and four composite cognitive z‐scores: (1) executive function (Trails B and Digit Symbol Substitution), (2) attention (Trails A and Digit Span Backward), (3) memory (Wechsler Memory Scale Logical Memory and Spanish English Verbal Learning Test), and (4) language (F‐A‐S and Animal Naming). We used linear regression models, adjusted for demographics and cognitive status (normal cognition vs. mild cognitive impairment (MCI) or dementia).

**Result:**

Of the 856 participants, 564 (65.9%) were women, 228 (26.6%) Black, 260 (30.4%) Mexican American, and 368 (43.0%) Non‐Hispanic White individuals. They had a mean age of 66.4±8.6 years, and 22% had MCI or dementia. Compared to older adults with normal cognition, participants with MCI/dementia had lower sleep efficiency, longer WASO, higher SFI, lower amplitude, mesor, and pseudo‐F, and a later acrophase (all *p* <0.005). After controlling for age, sex, race/ethnicity, education, and cognitive status, long sleep duration (>8 hours) was associated with poorer executive function (β=‐0.249, 95% confidence interval (CI)=‐0.355,‐0.143) and attention (β=‐0.160, 95% CI=‐0.273,‐0.047). Long WASO (higher tertile; >62.3 minutes) was linked to poorer executive function (β=‐0.187, 95% CI=‐0.298,‐0.075), attention (β=‐0.190, 95% CI=‐0.309,‐0.070), and memory (β=‐0.133, 95% CI=‐0.258,‐0.007). No associations were found for sleep efficiency, SFI, or RAR parameters.

**Conclusion:**

Long sleep duration and WASO were associated with deficits in cognitive domains, notably executive function and attention, underscoring the importance of domain‐specific assessments in sleep and cognitive research.

